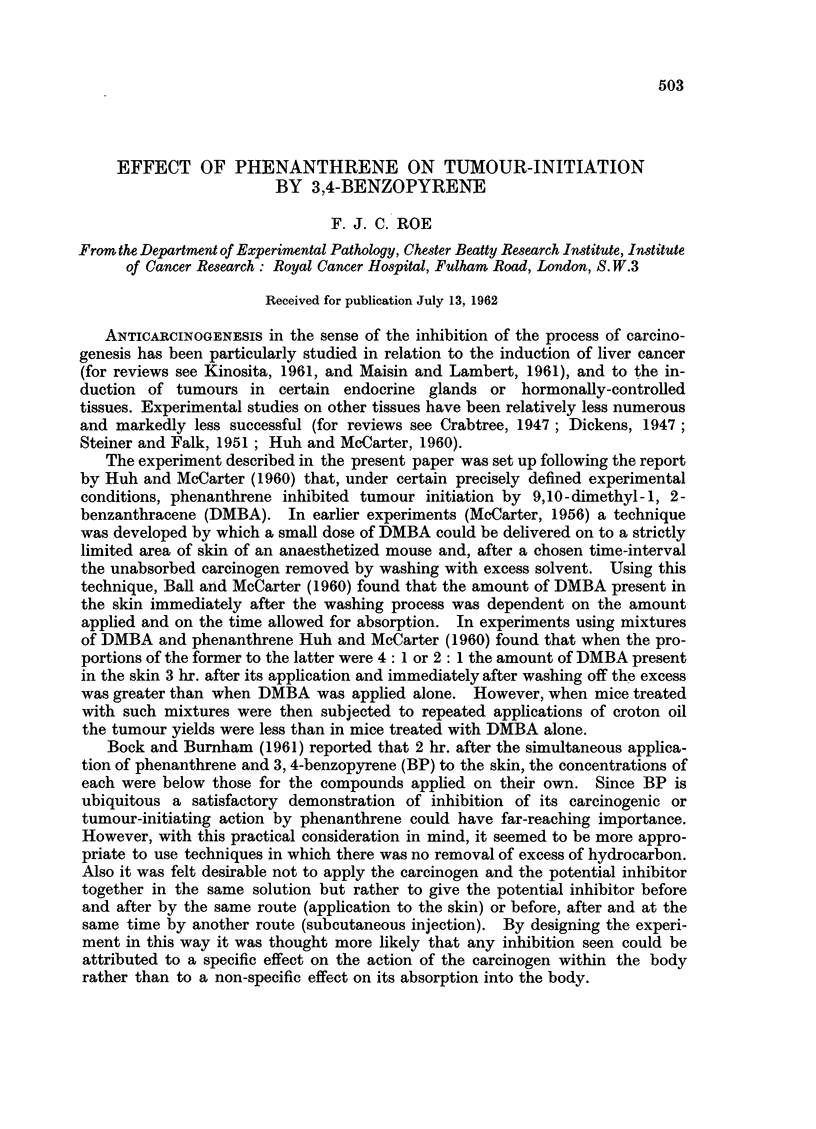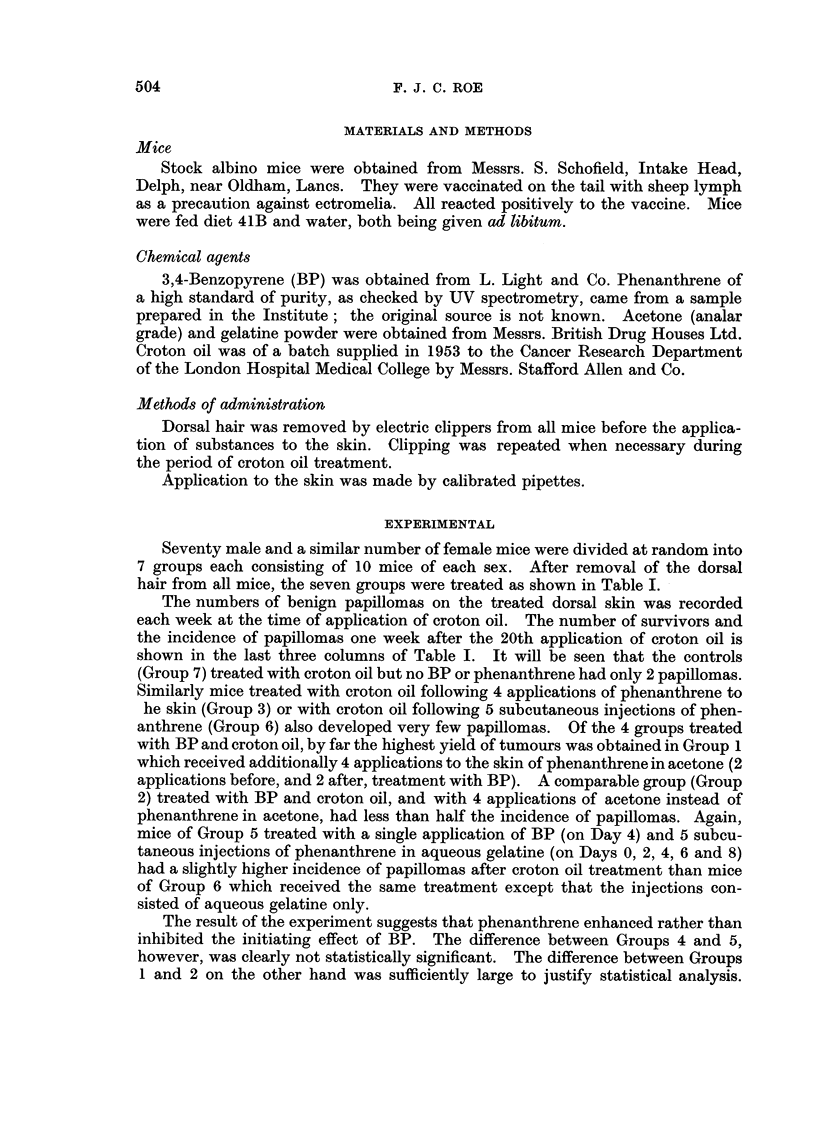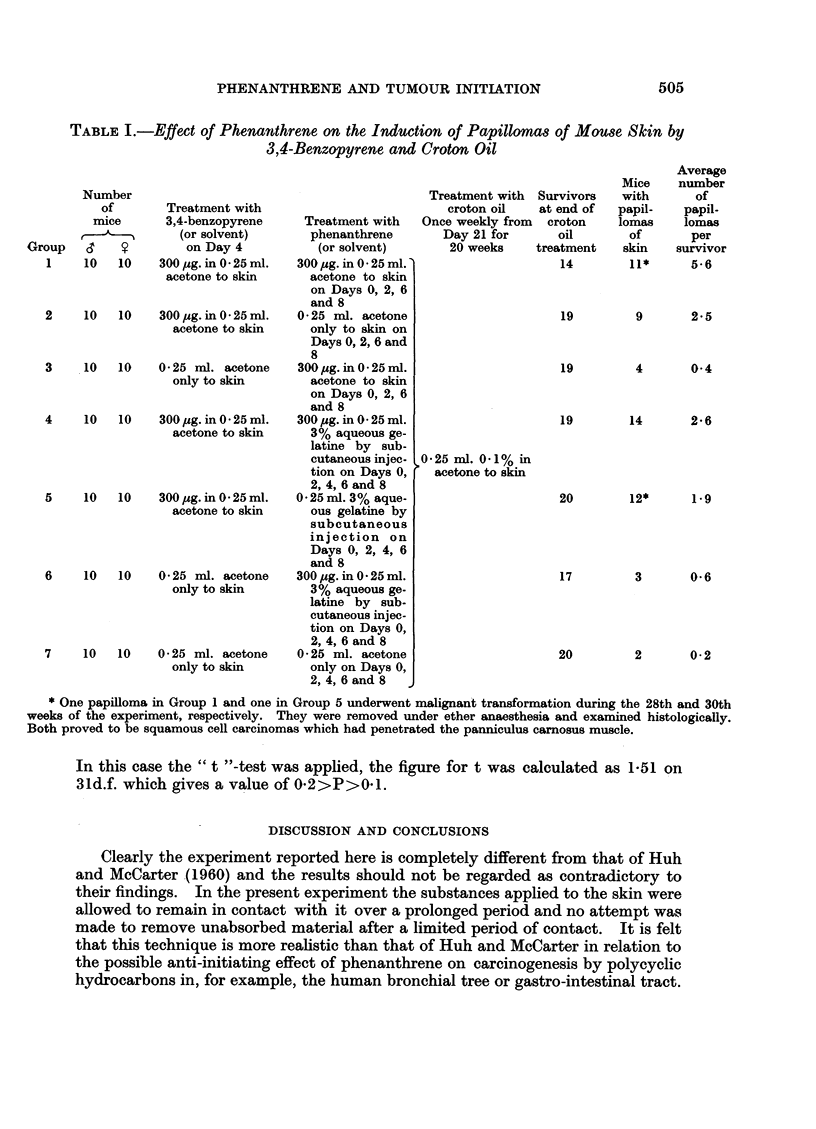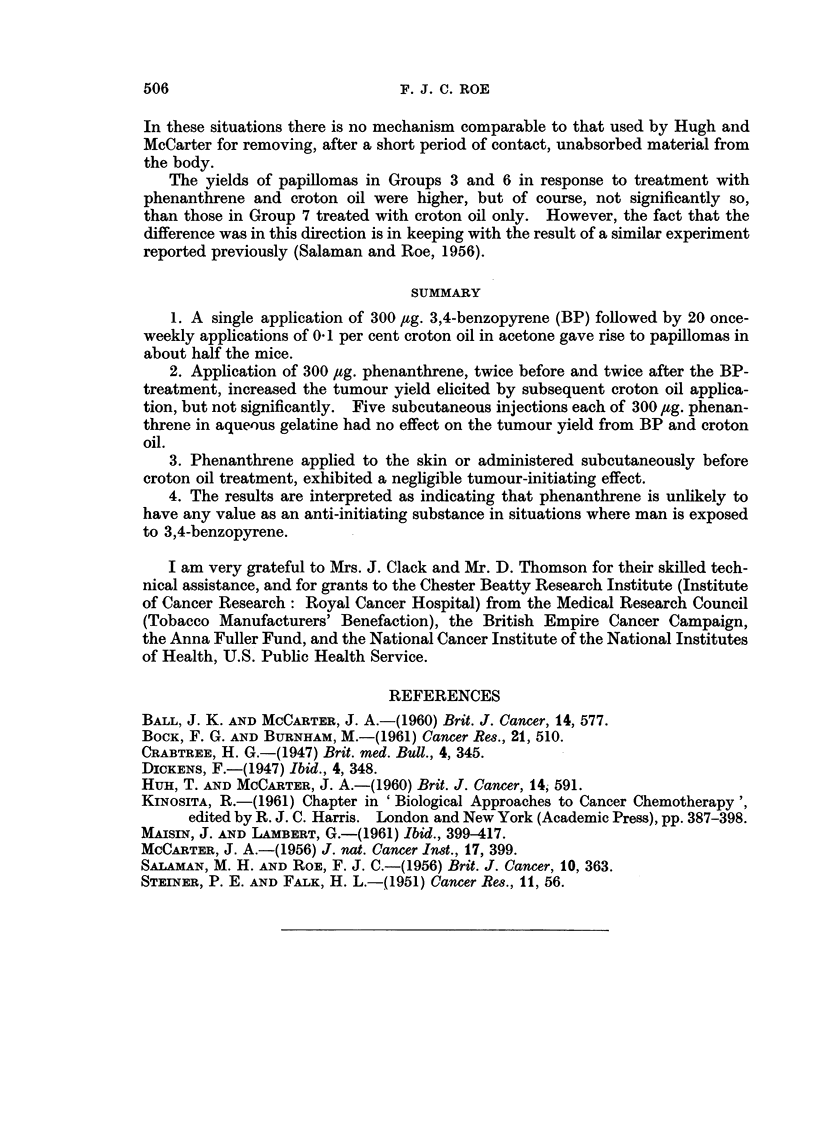# Effect of Phenanthrene on Tumour-initiation by 3,4-Benzopyrene

**DOI:** 10.1038/bjc.1962.58

**Published:** 1962-09

**Authors:** F. J. C. Roe


### EFFECT OF PHENANTHRENE ON TUMOUR-INITIATION BY 3,4-BENZOPYRENE

F. J. C. ROE

*From the Department of Experimental Pathology, Chester Beatty Research Institute, Institute of Cancer Research : Royal Cancer Hospital, Fulham Road, London, S.W.3*

Received for publication July 13, 1962

ANTICARCINOGENESIS in the sense of the inhibition of the process of carcinogenesis has been particularly studied in relation to the induction of liver cancer (for reviews see Kinosita, 1961, and Maisin and Lambert, 1961), and to the induction of tumours in certain endocrine glands or hormonally-controlled tissues. Experimental studies on other tissues have been relatively less numerous and markedly less successful (for reviews see Crabtree, 1947 ; Dickens, 1947 ; Steiner and Falk, 1951 ; Huh and McCarter, 1960).

The experiment described in the present paper was set up following the report by Huh and McCarter (1960) that, under certain precisely defined experimental conditions, phenanthrene inhibited tumour initiation by 9,10-dimethyl-1, 2-benzanthracene (DMBA). In earlier experiments (McCarter, 1956) a technique was developed by which a small dose of DMBA could be delivered on to a strictly limited area of skin of an anaesthetized mouse and, after a chosen time-interval the unabsorbed carcinogen removed by washing with excess solvent. Using this technique, Ball and McCarter (1960) found that the amount of DMBA present in the skin immediately after the washing process was dependent on the amount applied and on the time allowed for absorption. In experiments using mixtures of DMBA and phenanthrene Huh and McCarter (1960) found that when the proportions of the former to the latter were 4 : 1 or 2 : 1 the amount of DMBA present in the skin 3 hr. after its application and immediately after washing off the excess was greater than when DMBA was applied alone. However, when mice treated with such mixtures were then subjected to repeated applications of croton oil the tumour yields were less than in mice treated with DMBA alone.

Bock and Burnham (1961) reported that 2 hr. after the simultaneous application of phenanthrene and 3, 4-benzopyrene (BP) to the skin, the concentrations of each were below those for the compounds applied on their own. Since BP is ubiquitous a satisfactory demonstration of inhibition of its carcinogenic or tumour-initiating action by phenanthrene could have far-reaching importance. However, with this practical consideration in mind, it seemed to be more appropriate to use techniques in which there was no removal of excess of hydrocarbon. Also it was felt desirable not to apply the carcinogen and the potential inhibitor together in the same solution but rather to give the potential inhibitor before and after by the same route (application to the skin) or before, after and at the same time by another route (subcutaneous injection). By designing the experiment in this way it was thought more likely that any inhibition seen could be attributed to a specific effect on the action of the carcinogen within the body rather than to a non-specific effect on its absorption into the body.

### MATERIALS AND METHODS

### *Mice*

Stock albino mice were obtained from Messrs. S. Schofield, Intake Head, Delph, near Oldham, Lancs. They were vaccinated on the tail with sheep lymph as a precaution against ectromelia. All reacted positively to the vaccine. Mice were fed diet 41B and water, both being given *ad libitum*.

### *Chemical agents*

3,4-Benzopyrene (BP) was obtained from L. Light and Co. Phenanthrene of a high standard of purity, as checked by UV spectrometry, came from a sample prepared in the Institute ; the original source is not known. Acetone (analar grade) and gelatine powder were obtained from Messrs. British Drug Houses Ltd. Croton oil was of a batch supplied in 1953 to the Cancer Research Department of the London Hospital Medical College by Messrs. Stafford Allen and Co.

### *Methods of administration*

Dorsal hair was removed by electric clippers from all mice before the application of substances to the skin. Clipping was repeated when necessary during the period of croton oil treatment.

Application to the skin was made by calibrated pipettes.

### EXPERIMENTAL

Seventy male and a similar number of female mice were divided at random into 7 groups each consisting of 10 mice of each sex. After removal of the dorsal hair from all mice, the seven groups were treated as shown in Table I.

The numbers of benign papillomas on the treated dorsal skin was recorded each week at the time of application of croton oil. The number of survivors and the incidence of papillomas one week after the 20th application of croton oil is shown in the last three columns of Table I. It will be seen that the controls (Group 7) treated with croton oil but no BP or phenanthrene had only 2 papillomas. Similarly mice treated with croton oil following 4 applications of phenanthrene to he skin (Group 3) or with croton oil following 5 subcutaneous injections of phenanthrene (Group 6) also developed very few papillomas. Of the 4 groups treated with BP and croton oil, by far the highest yield of tumours was obtained in Group 1 which received additionally 4 applications to the skin of phenanthrene in acetone (2 applications before, and 2 after, treatment with BP). A comparable group (Group 2) treated with BP and croton oil, and with 4 applications of acetone instead of phenanthrene in acetone, had less than half the incidence of papillomas. Again, mice of Group 5 treated with a single application of BP (on Day 4) and 5 subcutaneous injections of phenanthrene in aqueous gelatine (on Days 0, 2, 4, 6 and 8) had a slightly higher incidence of papillomas after croton oil treatment than mice of Group 6 which received the same treatment except that the injections consisted of aqueous gelatine only.

The result of the experiment suggests that phenanthrene enhanced rather than inhibited the initiating effect of BP. The difference between Groups 4 and 5, however, was clearly not statistically significant. The difference between Groups 1 and 2 on the other hand was sufficiently large to justify statistical analysis.

TABLE I.—*Effect of Phenanthrene on the Induction of Papillomas of Mouse Skin by 3,4-Benzopyrene and Croton Oil*

| Group | Number of mice ♂ | Number of mice ♀ | Treatment with 3,4-benzopyrene (or solvent) on Day 4 | Treatment with phenanthrene (or solvent) | Treatment with croton oil Once weekly from Day 21 for 20 weeks | Survivors at end of croton oil treatment | Mice with papillomas of skin | Average number of papillomas per survivor |
|---|---|---|---|---|---|---|---|---|
| 1 | 10 | 10 | 300 μg. in 0·25 ml. acetone to skin | 300 μg. in 0·25 ml. acetone to skin on Days 0, 2, 6 and 8 | 0·25 ml. 0·1% in acetone to skin (all groups) | 14 | 11* | 5·6 |
| 2 | 10 | 10 | 300 μg. in 0·25 ml. acetone to skin | 0·25 ml. acetone only to skin on Days 0, 2, 6 and 8 | | 19 | 9 | 2·5 |
| 3 | 10 | 10 | 0·25 ml. acetone only to skin | 300 μg. in 0·25 ml. acetone to skin on Days 0, 2, 6 and 8 | | 19 | 4 | 0·4 |
| 4 | 10 | 10 | 300 μg. in 0·25 ml. acetone to skin | 300 μg. in 0·25 ml. 3% aqueous gelatine by subcutaneous injection on Days 0, 2, 4, 6 and 8 | | 19 | 14 | 2·6 |
| 5 | 10 | 10 | 300 μg. in 0·25 ml. acetone to skin | 0·25 ml. 3% aqueous gelatine by subcutaneous injection on Days 0, 2, 4, 6 and 8 | | 20 | 12* | 1·9 |
| 6 | 10 | 10 | 0·25 ml. acetone only to skin | 300 μg. in 0·25 ml. 3% aqueous gelatine by subcutaneous injection on Days 0, 2, 4, 6 and 8 | | 17 | 3 | 0·6 |
| 7 | 10 | 10 | 0·25 ml. acetone only to skin | 0·25 ml. acetone only on Days 0, 2, 4, 6 and 8 | | 20 | 2 | 0·2 |

* One papilloma in Group 1 and one in Group 5 underwent malignant transformation during the 28th and 30th weeks of the experiment, respectively. They were removed under ether anaesthesia and examined histologically. Both proved to be squamous cell carcinomas which had penetrated the panniculus carnosus muscle.

In this case the " t "-test was applied, the figure for t was calculated as 1·51 on 31d.f. which gives a value of $0{\cdot}2 > P > 0{\cdot}1$.

### DISCUSSION AND CONCLUSIONS

Clearly the experiment reported here is completely different from that of Huh and McCarter (1960) and the results should not be regarded as contradictory to their findings. In the present experiment the substances applied to the skin were allowed to remain in contact with it over a prolonged period and no attempt was made to remove unabsorbed material after a limited period of contact. It is felt that this technique is more realistic than that of Huh and McCarter in relation to the possible anti-initiating effect of phenanthrene on carcinogenesis by polycyclic hydrocarbons in, for example, the human bronchial tree or gastro-intestinal tract.

In these situations there is no mechanism comparable to that used by Hugh and McCarter for removing, after a short period of contact, unabsorbed material from the body.

The yields of papillomas in Groups 3 and 6 in response to treatment with phenanthrene and croton oil were higher, but of course, not significantly so, than those in Group 7 treated with croton oil only. However, the fact that the difference was in this direction is in keeping with the result of a similar experiment reported previously (Salaman and Roe, 1956).

### SUMMARY

1. A single application of 300 μg. 3,4-benzopyrene (BP) followed by 20 once-weekly applications of 0·1 per cent croton oil in acetone gave rise to papillomas in about half the mice.

2. Application of 300 μg. phenanthrene, twice before and twice after the BP-treatment, increased the tumour yield elicited by subsequent croton oil application, but not significantly. Five subcutaneous injections each of 300 μg. phenanthrene in aqueous gelatine had no effect on the tumour yield from BP and croton oil.

3. Phenanthrene applied to the skin or administered subcutaneously before croton oil treatment, exhibited a negligible tumour-initiating effect.

4. The results are interpreted as indicating that phenanthrene is unlikely to have any value as an anti-initiating substance in situations where man is exposed to 3,4-benzopyrene.

I am very grateful to Mrs. J. Clack and Mr. D. Thomson for their skilled technical assistance, and for grants to the Chester Beatty Research Institute (Institute of Cancer Research : Royal Cancer Hospital) from the Medical Research Council (Tobacco Manufacturers' Benefaction), the British Empire Cancer Campaign, the Anna Fuller Fund, and the National Cancer Institute of the National Institutes of Health, U.S. Public Health Service.

### REFERENCES

Ball, J. K. and McCarter, J. A.—(1960) *Brit. J. Cancer*, **14**, 577.
Bock, F. G. and Burnham, M.—(1961) *Cancer Res.*, **21**, 510.
Crabtree, H. G.—(1947) *Brit. med. Bull.*, **4**, 345.
Dickens, F.—(1947) *Ibid.*, **4**, 348.
Huh, T. and McCarter, J. A.—(1960) *Brit. J. Cancer*, **14**, 591.
Kinosita, R.—(1961) Chapter in 'Biological Approaches to Cancer Chemotherapy', edited by R. J. C. Harris. London and New York (Academic Press), pp. 387–398.
Maisin, J. and Lambert, G.—(1961) *Ibid.*, 399–417.
McCarter, J. A.—(1956) *J. nat. Cancer Inst.*, **17**, 399.
Salaman, M. H. and Roe, F. J. C.—(1956) *Brit. J. Cancer*, **10**, 363.
Steiner, P. E. and Falk, H. L.—(1951) *Cancer Res.*, **11**, 56.